# The Effects of Carbon Nanotubes on the Mechanical and Wear Properties of AZ31 Alloy

**DOI:** 10.3390/ma10121385

**Published:** 2017-12-04

**Authors:** Mingyang Zhou, Xiaoni Qu, Lingbao Ren, Lingling Fan, Yuwenxi Zhang, Yangyang Guo, Gaofeng Quan, Qi Tang, Bin Liu, Hao Sun

**Affiliations:** 1 Key Laboratory of Advanced Technologies of Materials, Ministry of Education, Chengdu 610031, China; 18782951516@163.com (M.Z.); xiaoniqu@163.com (X.Q.); 15928167496@163.com (L.R.); 18200100270@163.com (L.F.); zywx@my.swjtu.edu.cn (Y.Z.); 15108226903@163.com (Y.G.); tangqi@my.swjtu.edu.cn (Q.T.); 17308373813@163.com (B.L.); suns_h@163.com (H.S.); 2 School of Materials Science and Engineering, Southwest Jiaotong University, Chengdu 610031, China

**Keywords:** carbon nanotube, metal matrix nanocomposites, texture, strengthening mechanisms, wear behavior

## Abstract

Carbon nanotube (CNT)-reinforced AZ31 matrix nanocomposites were successfully fabricated using a powder metallurgy method followed by hot extrusion. The influence of CNTs on microstructures, mechanical properties, and wear properties were systematically investigated by optical microscope (OM), scanning electron microscope (SEM), X-ray diffraction (XRD), hardness test, tensile test, and wear test. The results revealed that the nanocomposites showed a slightly smaller grain size compared with the matrix and uniform distribution that CNTs could achieve at proper content. As a result, the addition of CNTs could weaken basal plane texture. However, the yield strength and ultimate tensile strength of the composites were enhanced as the amount of CNTs increased up to 2.0 wt. %, reaching maximum values of 241 MPa (+28.2%) and 297 MPa (+6.1%), respectively. The load transfer mechanism, Orowan mechanism, and thermal mismatch mechanism played important roles in the enhancement of the yield strength, and several classical models were employed to predict the theoretical values. The effect of CNT content on the friction coefficient and weight loss of the nanocomposites was also studied. The relationships between the amount of CNTs, the friction coefficient, and weight loss could be described by the exponential decay model and the Boltzmann model, respectively.

## 1. Introduction

The design of lightweight vehicles and aircrafts, is considered one of the most effective strategies for improving fuel efficiency and reducing anthropogenic climate change. As a result, this area of research has attracted an increasing amount of attention in recent years [1,2,3]. Moreover, the compelling need for lightweight materials is driving the development and motivating a wider spread application of magnesium (Mg) alloys, the ultra-lightweight materials with low density, a high strength-to-weight ratio, and superior damping capacity [1,4]. However, some inherent weaknesses, such as low absolute strength and stiffness, low wear resistance, and inferior creep resistance often restrict the scope of their applications. The addition of discontinuous reinforcements, especially nano-sized particles, into the magnesium matrix can significantly improve the physical, mechanical, and damping properties of magnesium alloys beyond the limits dominated by traditional alloying [5,6].

Carbon nanotubes (CNTs) have been considered an ideal reinforcement for metal matrix composites since Sumio Iijima discovered them in 1991, due to their excellent mechanical and physical properties [7,8]. The incorporation of a small amount of CNTs into the Mg matrix to form nanocomposites can significantly enhance mechanical properties [9,10]. Besides, with the development of the synthesis technology of CNTs, their price point is becoming more and more acceptable, making them available to be extensively used in Mg matrix nanocomposites [11]. These lightweight nanocomposites with good mechanical properties have huge potential in the aerospace, automobile, and transportation industries. Thus, it is essential to investigate CNT-reinforced Mg matrix nanocomposites.

Over the past few years, CNT-reinforced Mg matrix composites have received much attention, and several methods have been proposed to fabricate these composites with a uniform distribution of CNTs in the matrix, including powder metallurgy with ball milling [12], stir casting with ultrasonic vibration [13], disintegrated melt deposition technique (DMD) [14], infiltration method [15], and so on [16]. Many previous works have proved that a powder metallurgy method can incorporate more CNTs into the Mg matrix and achieve a more uniform distribution than stir casting and DMD [17,18]. What’s more, nanocomposites fabricated by powder metallurgy usually have superior mechanical properties than other methods due to their finer grains [17]. Shimizu et al. [19] demonstrated that a 1 wt. % short and straight CNT-reinforced AZ91 matrix fabricated by powder metallurgy could achieve a uniform distribution of CNTs and significantly enhance the tensile strength of the matrix (from 315 MPa to 388 MPa). Gupta et al. [11] fabricated Al+CNT-reinforced Mg nanocomposites using a powder metallurgy method involving microwave-assisted rapid sintering and hot extrusion, and achieved significant grain refinement and minimal porosity compared with monolithic Mg. Yuan et al. [20] coated magnesium oxide nanoparticles on the surface of CNTs to increase the interfacial bonding strength, and fabricated CNTs/MgO-reinforced AZ91 matrix nanocomposites using a powder metallurgy method. This way, both the yield strength and ductility of the composites were significantly enhanced. All of these research studies were focused on mechanical properties, especially strength and ductility, but wear property has never been studied. However, it is also essential for to examine wider applications for these magnesium matrix composites fabricated by powder metallurgy method [15].

As for strengthening mechanisms, the previous works generally considered (i) the load transfer mechanism, (ii) the generation of dislocations by the mismatch in the coefficient of thermal expansions (CTE) between the matrix and reinforcements, (iii) Orowan looping, and (iv) Hall–Petch strengthening by grain refinement [5,21,22,23].

The basis of the load transfer mechanism is the shear lag model, which was initially proposed by Kelly and Tyson [24]. The applied stress can be transferred from the matrix to reinforcements (e.g., CNTs) through interfacial shear stresses, and the resulting increase in the yield strength of the composites due to load transfer can be expressed as the following equation [20]:

(1) $$\Delta \sigma_{LT}=V_{CNT}\sigma_{ym}(\frac{l}{2d}-1)$$

where *σ_ym_* is the yield strength of the matrix, *l* is the average length of the CNTs, *d* is the average diameter of the CNTs, and *V_CNT_* is the volume fraction of the CNTs.

The increase in the yield strength of the composites caused by thermal mismatch depends on the difference in the CTE between the matrix and reinforcements; the higher the difference, the higher the increase of the strength that can be obtained. Geoge et al. [25] attributed the strength improvement to the “prismatic punching” of dislocations at the surface, which can promote the work hardening of the matrix. The improvements to the yield strength due to thermal mismatch can be written as [26]:

(2) $$\Delta \sigma_{TM}=\alpha G_{m}b\sqrt{\frac{12\Delta T\Delta CV_{CNT}}{bd}}$$

where *α* is a constant (1.25 in our case) [27], *G_m_* is the shear modulus of the matrix (1.66 × 104 MPa), *b* is the Burgers vector of the matrix (3.21 × 10^−10^ m) [28], Δ*T* is the range of temperature from tensile test to fabrication process (375 K), Δ*C* is the difference in CTE between the matrix and CNTs (the CET for Mg and CNTs is 2.5 × 10^−5^ K^−1^ and 1 × 10^−6^ K^−1^, respectively) [29]. *V_CNT_* and *d* are the same as defined earlier.

Orowan looping can significantly influence the strengthening of nanoparticle-reinforced metal matrix nanocomposites, because nano-sized CNTs can inhibit the dislocation motion, leading to the “dislocation bending” between the CNTs [30]. Zhan and Chen [31] first took the Orowan strengthening effect into account to propose an analytical model for predicting the yield strength of metal matrix nanocomposites, and found that the prediction was in good agreement with the experiment data reported in the literature. The increase of the composites caused by Orowan looping can be given by [20]:

(3) $$\Delta \sigma_{Orowan}=0.8MG_{m}b\sqrt{\frac{2V_{CNT}}{\pi d^{2}}}$$

where *M* is the Taylor factor (3.0 for Mg), and *G_m_*, *b*, *d*, and *V_CNT_* are the same as defined above.

The decrease of grain size can also enhance yield strength, according to the Hall-Petch relationship, and its contribution can be described by the following equation [21]:

(4) $$\Delta \sigma_{Hall-Petch}=K(d_{com}^{-1/2}-d_{alloy}^{-1/2})$$

where *d_com_* and *d_alloy_* are the average grain sizes of the composite and alloy, respectively, and *K* is the Hall–Petch coefficient of the Mg alloy (0.1 MPa m^1/2^).

However, some other factors, such as the CNTs’ distribution, texture, interfacial bonding, and porosity in composites fabricated by a powder metallurgy method, will influence the strength of the composites significantly, and thus should also be taken into account.

In this study, we examined AZ31 magnesium alloy matrix nanocomposites reinforced with varying amounts of CNTs that were fabricated by a powder metallurgy method followed by hot extrusion. The effects of the amount of CNTs on the microstructure, mechanical properties, and fracture characteristics of AZ31 matix nanocomposites were systematically investigated. The strengthening mechanisms were also discussed in detail. In addition, the relationships between the CNTs’ content, the friction coefficient, and the weight loss of the nanocomposites were studied, and the prediction equations were also established by nonlinear fitting.

## 2. Materials and Methods

### 2.1. Materials

AZ31 magnesium alloy with the composition of 3.0 wt. % Al and 1.0 wt. % Zn was used as the raw material, and was prepared by the metal powders of Mg, Al, and Zn, which were purchased from CHINO New Material Technology Co., Ltd., Zhuzhou, China. The specifications of these metal powders are shown in Table 1. The reinforcements, multi-walled CNTs (30–50 nm in outer diameter, 1–2 μm in length, and 98% purity) were supplied by Chengdu Organic Chemistry Co., Ltd., Chengdu, China.

### 2.2. Fabrication of AZ31–CNTs Composites

**Figure 1:**
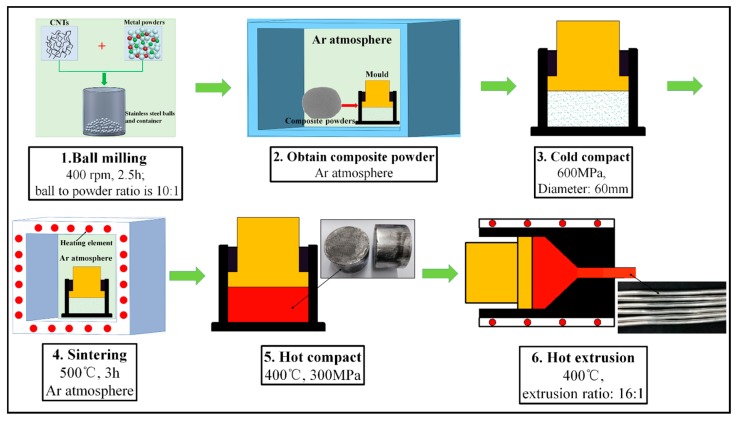
Sketch diagram of the fabrication process.

The composites and matrix were fabricated by powder metallurgy method (as shown in Figure 1). The nano-reinforcements (0.5 wt. %, 1.0 wt. %, 2.0 wt. %, and 4.0 wt. % CNTs, respectively) and 99 wt. % metal powders (AZ31) were added into a stainless steel container and mixed under the protection of argon atmosphere in a glove box to minimize the oxidation. The mixture was then milled in a PBM-4A planetary mill (ZOOKIN, Changsha, China) at 400 rpm for 2.5 h at room temperature using stainless steel balls of 5 mm and 10 mm as the grinding medium, and a ball to powder ratio of 10:1. The planetary mill was turned off for 8 m after every 20 m of work to prevent overheating. To avoid the excessive cold welding of the powders during milling, 0.3 wt. % of stearic acid of analytical reagent grade purity was added into the container as the process control agent. The milled mixture powders were taken out and put into a cylindrical steel die of 60 mm inner diameter in the glove box. The powders were then cold compacted by slowly increasing the pressure up to 600 MPa, and sintered for 3 h at 500 °C under the protection of argon atmosphere without demold. After the temperature of the die decreased to 400 °C, the compacts were hot pressed at a pressure of 300 MPa to obtain the resulting compacts with 45 mm height and 60 mm diameter. Finally, all of the resulting compacts were extruded at 400 °C with an extrusion ratio of 16:1 to obtain rod samples with a diameter of 15 mm. For comparison, the AZ31 matrix samples were prepared by the same conditions.

### 2.3. Characterization

The density of the polished samples was measured based on the Archimedean principle using an electronic density meter (ESJ182-4, Longteng, Shenyang, China) with an accuracy of 0.1 mg, and distilled water as an immersion fluid. Three samples were randomly selected from extruded rods and weighed both in air and while immersed in distilled water. Theoretical densities of the samples were calculated using the rule-of-mixture principle and assuming that there was no Mg/Al–CNTs interfacial reaction. The phases of the samples were examined by X-ray diffraction (XRD, PANalytical, B.V., Almelo, Holland) using Cu Kα radiation for a 10~90° range (scan step size 0.033°, time per step 12 s, and scan type was continuous). The samples were cut from the extrusion bars, ground on the 2000# SiC abrasive papers, polished to a mirror-like finish, and etched using 4% nitric acid alcohol solution. The microstructure of the samples was characterized using an optic microscope (OM, ZEISS, Oberkochen, Germany) and a scanning electron microscope (SEM, FEI, Hillsboro, OR, USA). The average grain size, G, was calculated using intercept line methods (G = L × 1.73; L, the average length of intercept line). Three tensile specimens with a diameter of 5 mm and a gauge length of 25 mm were machined from each material by electro-discharge cutting parallel to the extrusion direction. The tensile tests were carried out on an MTS-CMT5105 universal testing machine (SANS, Shenzhen, China) at room temperature with an initial strain rate of 0.001 s^−1^ (according to ASTM: E8/E8M-11 standards). The hardness was tested using Vickers hardness tester (Aiceyi, Dongguan, China) at a load of 1000 gf with a dwell time of 15 s. The wear behavior of the matrix and composites were investigated by using ball-on disk-wear test equipment (CETR UMT-2, Wode, Beijing, China) under air and dry sliding conditions. A high-carbon chromium-bearing steel ball with 10 mm diameter and a hardness of HRC57 was used as the counter material. The tests were conducted under the applied loads of 5 N, 10 N, and 50 N, respectively. The linear reciprocating frequency, displacement amplitude, and friction time were 0.5 HZ, 1000 μm, and 40 min, respectively. The test samples with 15 mm diameter and 5 mm height were cut from the extrusion bars, ground, polished, and cleaned using ultrasonic cleaning in ethanol before the test. Mass loss was measured with an electronic density meter (ESJ182-4, Longteng, Shenyang, China) with an accuracy of 0.1 mg. The coefficients of friction were obtained by measuring the friction torque between the ball and the disk. Three samples were tested for each load and material, and the average of three readings was considered as the results of weight loss and the coefficients of friction.

## 3. Results and Discussions

### 3.1. Density Measurements

The theoretical and experimental densities of the as-extruded AZ31 and its nanocomposites are listed in Table 2. It can be seen that the measured experimental density of AZ31 was slightly lower than the theoretical density, indicating a good diffusion of metal atoms at this sintering temperature, and that the parameters employed in cold compaction and hot extrusion were feasible. However, the porosity in all of the nanocomposites was much higher than that in the matrix. What’s more, the porosity level of the nanocomposites increased as the weight percentage of the CNTs increased, and the porosity level remained at about 2.5% when the CNTs addition increased from 0.5 wt. % to 2.0 wt. %. Besides, when the CNTs addition was 4.0 wt. %, the porosity increased dramatically up to 5.3%, which may be caused by the agglomeration of CNTs [32].

### 3.2. X-ray Analysis

**Figure 2:**
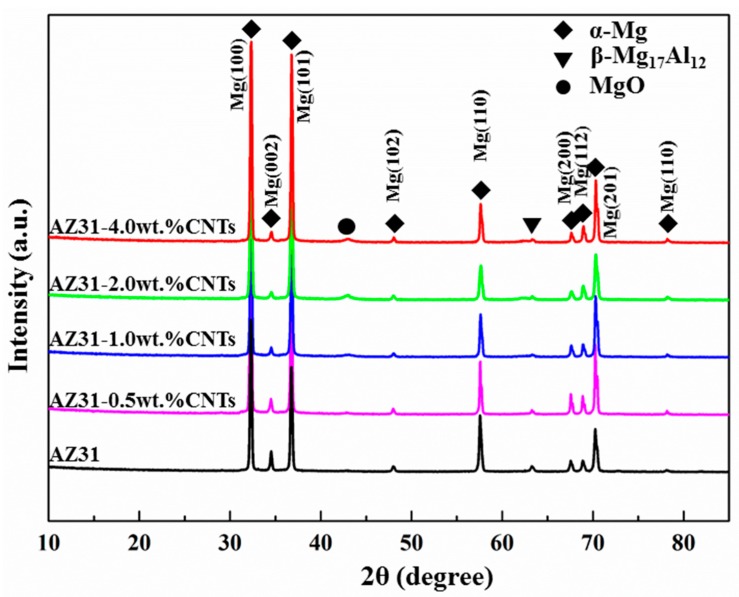
XRD (X-ray diffraction) patterns (taken perpendicular to the extrusion direction) of the AZ31 magnesium alloy and its nanocomposites.

The X-ray diffraction patterns of the as-extruded AZ31 and its nanocomposites taken perpendicular to extrusion are shown in Figure 2, and the texture results based on the X-ray diffraction are listed in Table 3. The diffraction peaks were analyzed using standard XRD procedures to determine phase components. It is seen that all of the samples contain α-Mg and β-Mg_17_Al_12_ phases, and no X-ray diffraction patterns of CNTs is observed in all of the nanocomposites. However, the existence of CNTs can be confirmed by comparing the peak intensities of α-Mg with different crystallographic planes. In addition to the α-Mg and β-Mg_17_Al_12_ phases, the MgO phase was also detected in all of the specimens, which likely resulted from in situ reactions between Mg and stearic acid, or between Mg and oxygen contamination on the surface of the metal powders and CNTs [33]. Besides, the intensity of the MgO peak increased as the weight percentage of the CNTs increased, which indicates that the zone with clusters of CNTs can be more easily oxidized. The formation of aluminum carbide was observed in previous research [34]. The absence of peaks of Al/Mg carbide phases in the XRD patterns may be attributed to the detection limit of the X-ray diffraction equipment [35]. Whether Al/Mg carbide phases formed in the composites needs to be further studied by transmission electron microscope (TEM).

It can be observed from Table 3 that with the addition of CNTs, the normalized peak intensities of the basal {0002} plane decreased, indicating a weakening of basal texture. These results are consistent with other reported texture results of Mg matrix nanocomposites that were fabricated by a powder metallurgy method, followed by extrusion [36,37]. In general, samples with weaker basal textures contained more grains with soft orientation, leading to an easier basal slip in the early stages of yielding and decreasing yield strength (0.2% proof stress) during the tensile test [38]. Therefore, the weakening basal texture may compensate for the strengthening effect of the nano-reinforcements in the matrix to some extent.

### 3.3. Microstructure Characterization

**Figure 3:**
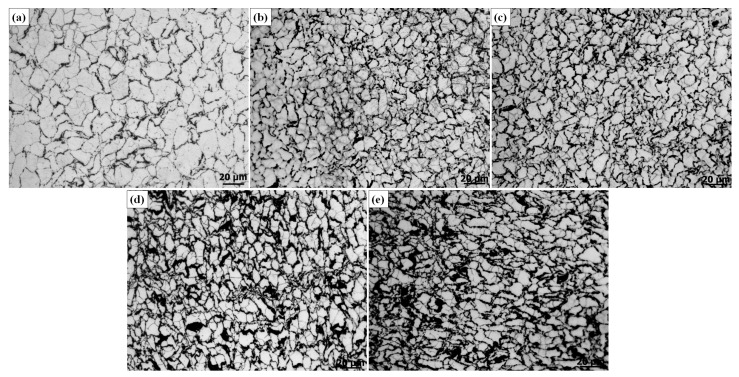
OM (optical microscope) graph of the as-extruded AZ31 and its nanocomposites: (**a**) AZ31, (**b**) AZ31-0.5 wt. % CNTs (Carbon nanotubes), (**c**) AZ31-1.0 wt. % CNTs, (**d**) AZ31-2.0 wt. % CNTs, (**e**) AZ31 wt. % CNTs.

**Figure 4:**
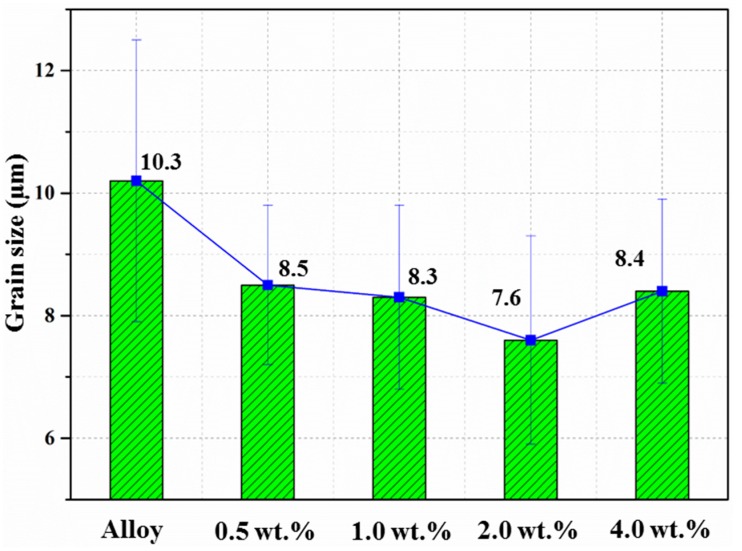
Grain size of the as-extruded AZ31 and its composites.

Figure 3 illustrates the OM graphs of the as-extruded AZ31 matrix and its nanocomposites, and Figure 4 shows the average grain size of the matrix and composites with different weight percentages. Adding CNTs to the matrix had a marginal grain refinement effect (about 20% reduction) in this study. Besides, with an increase of the amount of CNTs from 0.5 wt. % to 2.0 wt. %, the grain size of the composites gradually decreased, but the reduction degree was pretty limited. The refinement of grains can be attributed to two reasons: (a) the larger strain due to the addition of CNTs in the particle bands can promote the occurrence of dynamic recrystallization (DRX) during the extrusion; (b) CNTs distributing on the grain boundaries can significantly inhibit the grain boundaries’ migration during the sintering and extrusion processes [21]. However, with a further increase of the content of CNTs up to 4.0 wt. %, a large black area can be observed on the grain boundaries, which may be attributed to the clusters of CNTs [20]. Besides, the clusters of CNTs not only slightly compensate for the refinement of grains on the matrices, they also impede the densification of the composites, resulting in the deterioration of the mechanical properties of the composites [20].

**Figure 5:**
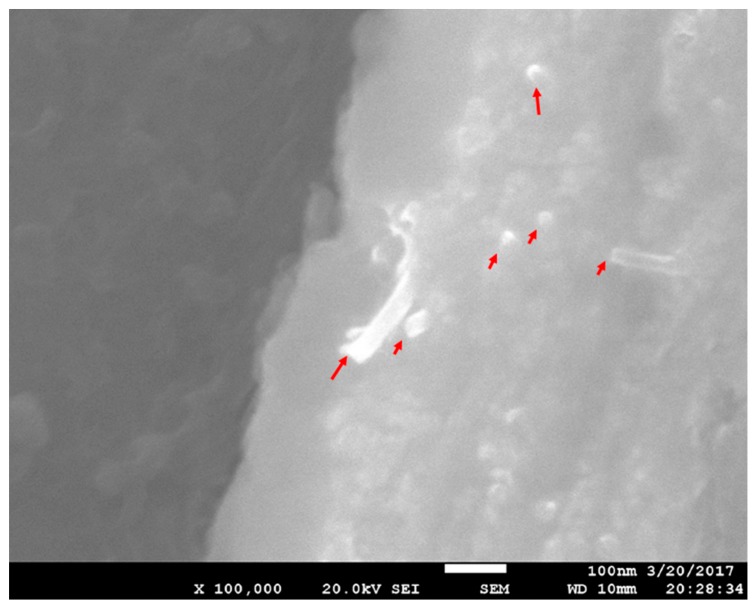
SEM (scanning electron microscope) images of the CNT distribution in the as-extruded composites with 1 wt. % CNTs.

Figure 5 shows a SEM image of homogeneously dispersed CNTs in the as-extruded composite with the addition of 1 wt. % CNTs. Individual CNTs (red arrows) were observed, and the clusters were not observed, which indicates that the CNTs distributed uniformly in the composites. Therefore, this method can fabricate magnesium matrix nanocomposites with uniform CNTs.

### 3.4. Mechanical Behavior

**Figure 6:**
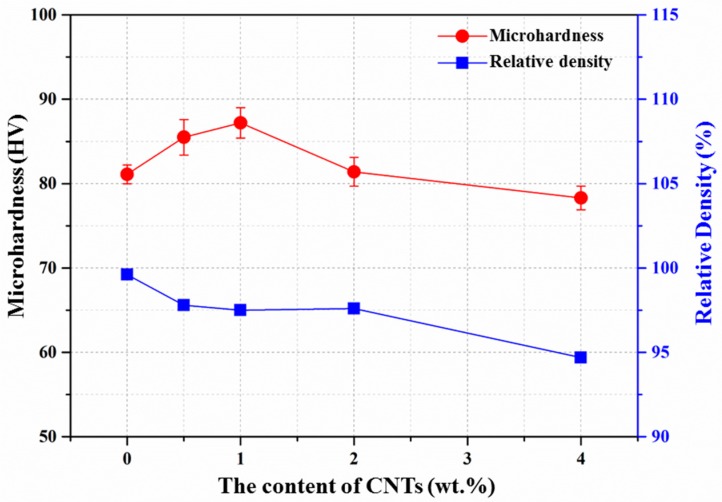
Curves of microhardness and relative density with the addition of varying amounts of CNTs.

**Figure 7:**
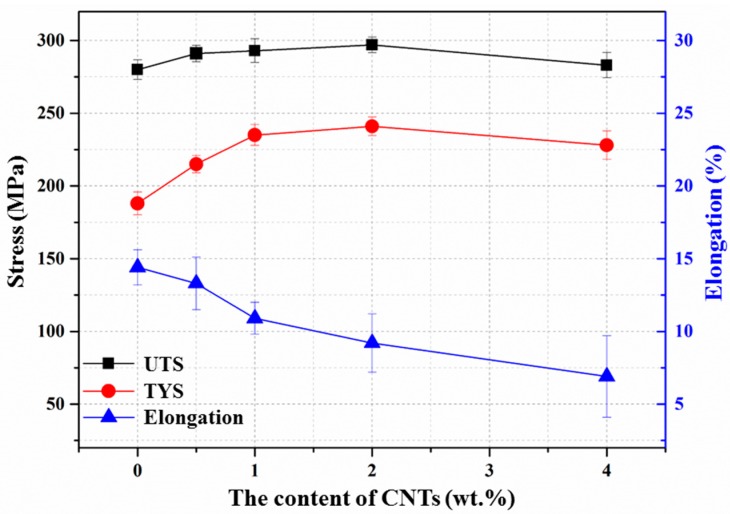
Effect of the addition of varying amounts of CNTs on the mechanical properties of the composites.

Figure 6 and Figure 7 show the effect of the addition of CNTs on the mechanical properties of the composites. The mechanical properties of the as-extruded AZ31 matrix and its nanocomposites are listed in Table 4. As shown in Figure 6, the microhardness (HV) of the alloy increased with the increasing amount of CNTs, and reached a maximum value of HV = 87.2 ± 1.8 for 1.0 wt. %. However, the microhardness decreased beyond 1.0 wt. % of CNTs. The increase of the microhardness can be attributed to: (i) grain refinement [39]; (ii) the high hardness and toughness of CNTs [36]; (iii) the higher constraint to localized matrix deformation during indentation, because of the presence of homogeneously dispersed nanoparticles in the matrix [32]. However, the decrease in microhardness can be explained by two reasons: the first is the presence of an agglomeration of CNTs in the composites, which can be observed in Figure 3d,e; the other is the lower densification (greater amount of micropores) of the composites, which leads to a deterioration of the microhardness to some degree.

The tensile yield strength (TYS), ultimate tensile strength (UTS), and elongation of the composites with different amount of CNTs are shown in Figure 7. It can be observed that the addition of CNTs significantly enhanced the TYS and slightly increased the UTS of the AZ31 matrix, which reached a maximum value of 241 MPa (+28.2%) and 297 MPa (+6.1%), respectively, with the addition of 2.0 wt. % CNTs. Both parameters dropped when the weight fraction of CNTs exceeded 2.0 wt. %, which might be caused by the formation of CNT clusters in the matrix. As reported in previous works, these clusters of CNTs can prevent effective bonding between the matrix and CNTs and lead to minute cracks [21], which can cause an increase in the porosity in the matrix [32], leading to the failure of the composites with lower strength. The elongation of the composites decreased gradually with as the amount of CNTs increased; however, the reduction level is much smaller than that in micrometer ceramics-reinforced metal matrix composites [39,40].

### 3.5. Strengthening Mechanisms

As mentioned above, the effect of CNTs on the grain refinement in this work is marginal, so the strengthening effect due to grain refinement can be ignored. The significance of each strengthening mechanism in the total improvement of the yield strength of the composites were mainly assessed based on two different models: (i) the linear model [27], and (ii) the root mean square model [22], and the two models can be expressed by following two equations, respectively:

(5) $$\Delta \sigma_{yc-Total}=\Delta \sigma_{LT}+\Delta \sigma_{TM}+\Delta \sigma_{Orowan}$$

(6) $$\Delta \sigma_{yc-Total}=\sqrt{{\left(\Delta \sigma_{LT}\right)}^{2}+{\left(\Delta \sigma_{TM}\right)}^{2}+{\left(\Delta \sigma_{Orowan}\right)}^{2}}$$

where Δ*σ_yc-Total_* is the total improvement in the yield strength of the composites. Thus, the theoretical yield strength of the composites (*σ_yc-Total_*) can be expressed as follows:

(7) $$\sigma_{yc-Total}=\sigma_{ym}+\Delta \sigma_{yc-Total}$$

**Figure 8:**
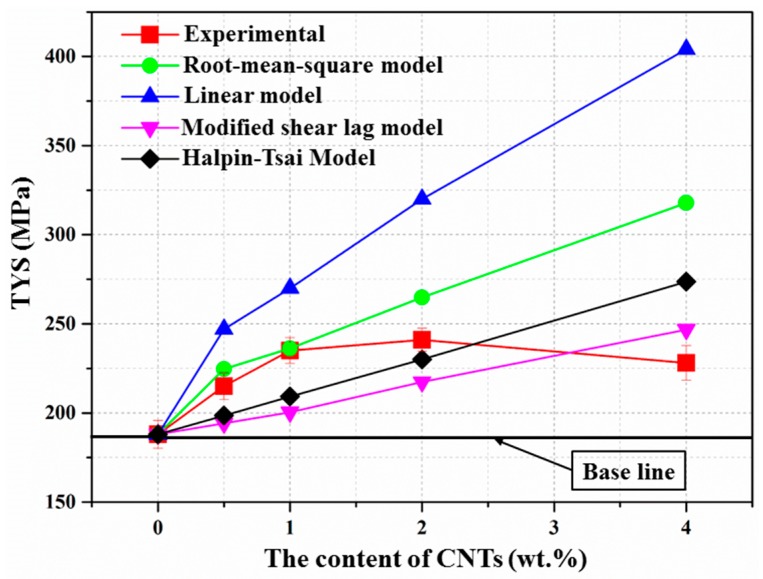
Plots of yield strength of the composites theoretically predicted by different models and experimentally obtained in this study.

The two models mentioned above, the modified shear-lag model and the Halpin–Tsai model were employed in this study to calculate the theoretic yield strength of the composites with different CNT contents, and the results are shown in Figure 8. The modified shear-lag model [41] mainly considers the load transfer mechanism and the effect of fiber orientations. The modified Halpin–Tsai model [16] proposes a semi-empirical description of short fiber-reinforced composites using the rule of mixture for discontinuous reinforcement, which is specified for metal matrix composites. The comparison of the experimental values and theoretically calculated values of the yield strength based on the models mentioned above are plotted in Figure 8. It can be observed that the experimental values are well matched with the root mean square model. The calculated yield stress is in good agreement with the experimental values at low CNT additions, and the discrepancy between the experimental and theoretical results are very small. However, the discrepancy increases dramatically with the increase of the CNTs content beyond 2.0 wt. %, which may be attributed to clusters of CNTs and higher porosity [20]. From Figure 8, it can also be found that the experimental values are even higher than the theoretical values calculated from the modified shear-lag model and the Halpin–Tsai model, which can predict the yield strength of the CNT-reinforced Al matrix well [42,43]. This might be because these two models mainly consider the load transfer mechanism, which significantly depends on the effective bonding between the matrix and reinforcements, but there is no obvious reaction between Mg and CNTs in Mg matrix composites [13,44]. This indicates that not enough interfacial carbide is formed during the fabricating process. However, it has been confirmed that interfacial carbide played a significant role in determining the load transfer efficiency of CNT-reinforced Al matrix composites [45], so load transfer can not play the completely dominant strengthening role in CNT-reinforced Mg matrix composites, and thermal mismatch and Orowan looping mechanisms cannot be ignored.

As for the linear model, the theoretical values are much higher than all of the experimental values, and the discrepancies increased as the amount of CNTs increased, which indicates that the strengthening effects were overestimated in this model. The discrepancies may be caused by the following reasons: (i) relatively low load transfer efficiency due to the weak interfacial bonding, as well as the effective CNTs volume fraction that could actually transfer the load being lower than the total volume fraction because of the existence of CNT clusters [42], (ii) a part of the CNTs were distributed along the grain boundaries, which could weaken the Orowan looping strengthening [46], (iii) the weakened basal texture mentioned above might soften the composites, which has been confirmed by previous works [38], (iv) the pores with irregular shapes could lead to high stress concentrations and pores located at the interfaces between CNTs and matrix could decrease the effective bonded area at the interfaces, which also reduced the load transfer efficiency [42]. Besides, the effect of pores on the strength of the materials fabricated by powder metallurgy can be expressed by the empirical equation [47]:

(8) $$\sigma_{P}=\sigma_{0}\exp(-\lambda \theta )$$

where *σ_P_* is the strength of the composites with pores, *σ*_0_ is the strength of the composites without pores, *θ* is the volume fraction of porosity, and *λ* is a constant for all of the materials with porosity.

In order to obtain a more precise prediction model, all of the factors that might influence the strengthening effects should be taken into account. Therefore, further work is needed to: reveal the effective load transfer coefficient for CNT-reinforced metal matrix composites with no obvious reaction between CNTs and the matrix, quantify an effective volume fraction for load transfer and Orowan looping, and confirm the constant (*λ*) for magnesium alloys.

For the CNT-reinforced metal matrix composites, the tensile strength of the composites is strongly influenced by the CNTs’ length, and is always theoretically calculated by the Kelly–Tyson formula [21], which can be expressed as:

(9) $$\sigma_{c}=V_{CNT}\times \sigma_{CNT}(\frac{l}{2l_{c}})+V_{m}\times \sigma_{m}$$

where *σ_c_* and *σ_m_* are the tensile strength of the composite and the matrix, respectively; *σ_CNT_* is the strength of the CNTs; *V_m_* is the volume fraction of the matrix; and *l_c_* is the critical length of the CNTs, which can be calculated by the following equation [48]:

(10) $$l_{c}=\frac{\sigma_{CNT}\times d}{\sigma_{m}}$$

**Figure 9:**
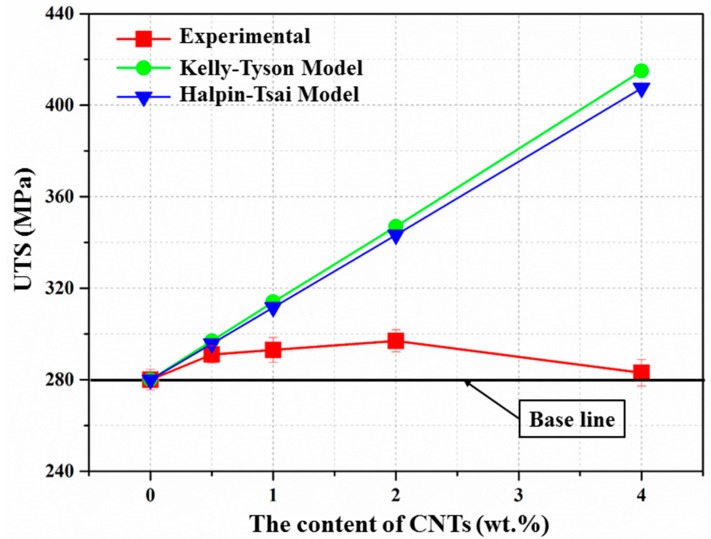
Plots of the ultimate tensile strength (UTS) of the composites theoretically predicted by different models and experimentally obtained in this study.

All of the variables are the same as defined above. From Equation (9), the calculated lc in this work is about 4.3 μm, and the average length of the CNTs used in this study is 1.25 μm, indicating that the calculated stress on the CNTs cannot reach the fracture strength of the CNTs, and Equation (8) can be used to predict the strength of the composites. The theoretically predicted and experimentally obtained tensile strength of the composites are shown in Figure 9. It can be observed that the theoretical values are in good agreement with the experimental values at low CNT content; however, the discrepancies increase as the amount of CNTs incresae. This may be caused by the agglomerations of the CNTs, which influence load transfer significantly. Besides, the Halpin–Tsai model was also employed to calculate the theoretical tensile strength of the composites, and it presents nearly the same trend as the Kelly–Tyson model.

### 3.6. Fracture Surface Characterization

**Figure 10:**
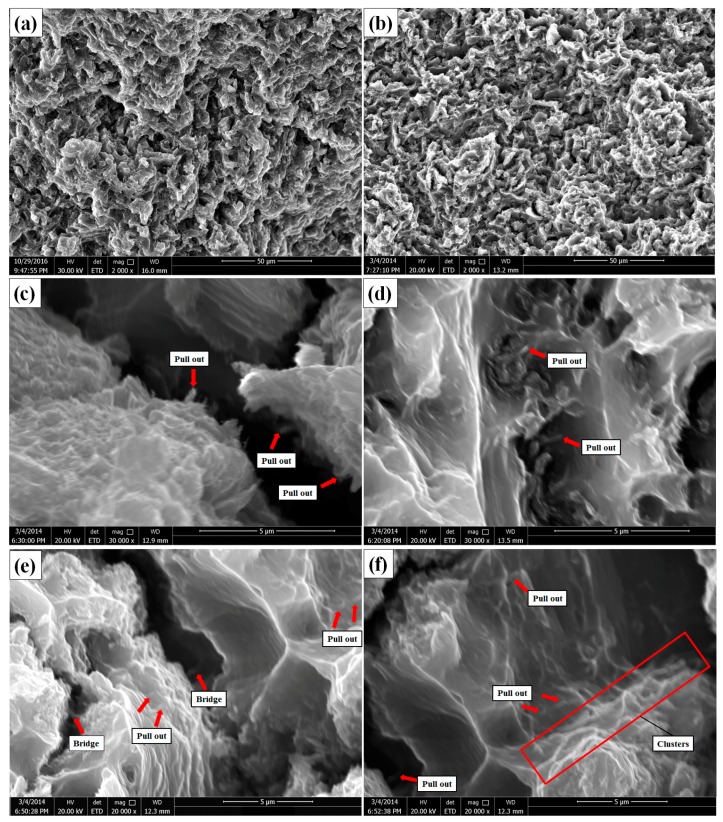
SEM images of the fracture surfaces of the as-extruded AZ31 composites and its nanocomposites: (**a**) AZ31, (**b**) AZ31-1.0 wt. % CNTs, (**c**) AZ31-0.5 wt. % CNTs, (**d**) AZ31-1.0 wt. % CNTs, (**e**) AZ31-2.0 wt. % CNTs and (**f**) AZ31-2.0 wt. % CNTs ((**a**,**b**) are low magnification images, and (**c**–**f**) are high magnification images).

Figure 10 illustrates the fracture surface of the as-extruded AZ31 matrix and its nanocomposites after tensile fracture. It can be observed that the cleavage steps and dimples are present both in monolithic AZ31 and its nanocomposite in Figure 10a,b, indicating both the brittle and ductile fractures that occurred during the tensile test. From Figure 10c–f, some individual CNTs are pulled out from the matrix (red arrows) and exposed outside the fracture surface, indicating that the load transfer was operative [20]. Besides, bridge phenomena can also be observed when the addition of CNTs is 2.0 wt. % (as seen in Figure 10e), which can further increase the strength of the composite by improving load transfer efficiency. The matrix would fracture firstly during the test, while the ultra-high strength CNTs could improve their load tolerance if they were dispersed uniformly in the matrix. As the load further increased, it would be transferred to the interface of the reinforcements and matrix, and the reinforcements would be pulled out from the matrix as the microcracks grew (Figure 10c–f) [49]. In addition, CNT clusters can also be observed in the fracture surface when the amount of CNTs reached 4.0 wt. %, as seen in Figure 10f (red box). These clusters of CNTs can significantly deteriorate the mechanical properties of the composites, which can be proved by the mechanical properties tested in our study.

### 3.7. Wear Behavior

**Figure 11:**
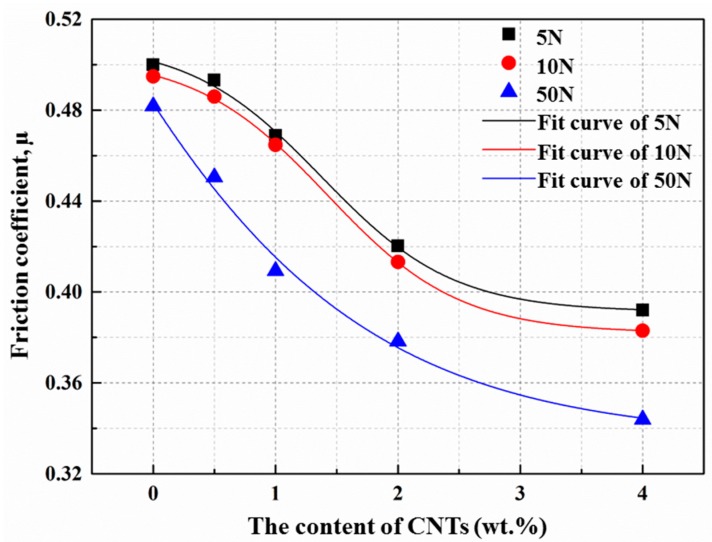
Friction coefficient variation as a function of CNTs content in the as-extruded AZ31 matrix at different normal loads.

Figure 11 shows the variation of the friction coefficient (μ) of the composites with different CNTs content at three different applied loads. It can be observed that the friction coefficient decreases with an increase of the content of CNTs at the same load condition; however, the decrease extent reduces when CNTs content is beyond 2.0 wt. %. This may be attributed to the self-lubrication effect of CNTs. When they were pulled out from the matrix during the wear tests, they could contact the counter material directly and decrease the contact area between the matrix and the counter material, leading to a decrease of the friction coefficient [50]. Besides, it has been proven in previous research [51,52,53] that the formation of carbon film could cover the wear surface and act as a solid lubricant that decreases the coefficient of friction. However, the lubrication effect was weakened when the addition of CNTs exceeded 2.0 wt. %, which might be caused by the agglomeration of CNTs and higher porosity. From Figure 11, it can also be observed that with the increase of the applied load at the same CNTs addition, the friction coefficient decreases. This might be attributed to the increase in ploughing force and penetration inside the samples at higher applied load conditions, which can generate more pull-out of CNTs from the matrix, leading to the improvement of the lubrication efficiency of the carbon film. What’s more, according to the distribution of the points in Figure 11, the Boltzmann model was adopted to fit the curves of the friction coefficient versus the content of CNTs, and the R-Square of the regression equations (as shown in Table 5) are nearly 0.99, indicating that the relationship between the friction coefficient and the content of CNTs can be well described by the Boltzmann model.

**Figure 12:**
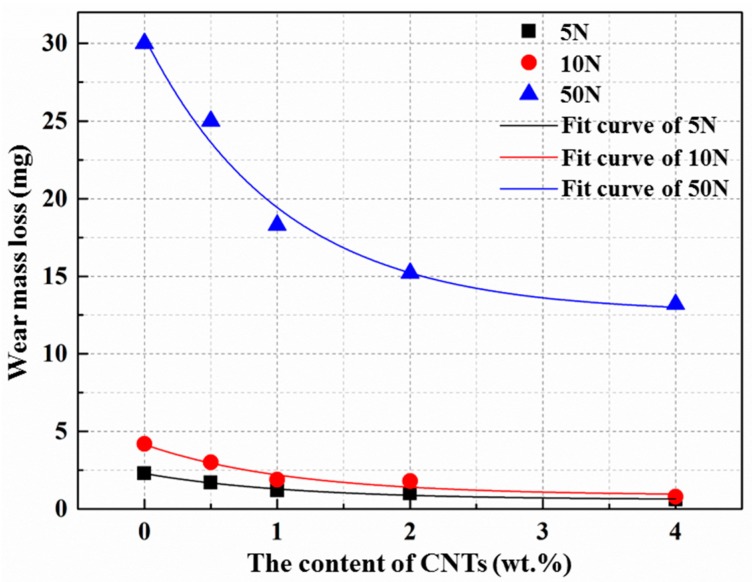
Wear mass loss variation as a function of CNT content in the as-extruded AZ31 matrix at different normal loads.

Figure 12 illustrates the effect of the amount of CNTs on the wear mass loss of the as-extruded AZ31 matrix at different loads. It can be observed that the weight loss of the composites decreases with as the amount of CNTs increases, and increases significantly with the increase of applied load values in this work. The decrease in the weight loss can be attributed to two main reasons: (i) the addition of CNTs can enhance and improve the hardness and strength of the matrix, which can improve its plastic deformation resistance and weaken the ploughing effect of the counter material on the matrix, leading to a lower weight loss; (ii) the self-lubrication effect of CNTs corresponds to a lower friction coefficient of the composites in the matrix, which can also help decrease the weight loss [50]. As for the influence of the load, the ploughing effect increases with the increase of the load value, leading to the increase of the weight loss. Further, the reduction level of the weight loss at high load conditions is much higher than that at low load conditions, which indicates that CNTs play a more significant role in reducing the weight loss for the composites at a high-applied load level. This may be because more pulled-out CNTs can be brought into the friction surface due to a higher ploughing force at higher load conditions, resulting in a better weight loss reduction effect. Besides, the weight loss of the composites gradually becomes stable when the addition of CNTs exceeds 2.0 wt. %, which may be caused by the formation of the agglomerations of CNTs and more pores. These agglomerations and pores can not only weaken the strengthening effect, they can also reduce the self-lubrication effect. What’s more, according to the distribution of the points in Figure 12, the exponential decay model was adopted to fit the curves of the friction coefficient versus the content of CNTs, and the R-Square of the regression equations (as shown in Table 6) are nearly 0.97, which means that this model can be used to predict weight loss during the CNTs content range between 0.0 to 4.0 wt. % in this work.

## 4. Conclusions

In summary, AZ31 alloy and CNT-reinforced AZ31 matrix nanocomposites were successfully fabricated via metallurgy method followed by hot extrusion. The influence of CNTs on microstructures, mechanical properties, and the wear behavior of the nanocomposites has been investigated. Based on the analysis of the results, conclusions can be drawn as follows:

(1) The powder metallurgy method could successfully fabricate CNT-reinforced AZ31 matrix nanocomposites with uniform distribution of the nano-reinforcements at proper content. Adding CNTs into AZ31 matrix could also marginally decrease grain size.
(2) The addition of CNTs could weaken the basal plane texture of the AZ31 fabricated via powder metallurgy method followed by hot extrusion, which might counteract the strengthening effect of CNTs to some extent.
(3) Compared with monolithic AZ31, the yield strength, ultimate tensile strength, and microhardness increased by adding CNTs into the matrix. The yield strength and ultimate tensile strength reached a peak value of 241 MPa (+28.2%) and 297 MPa (+6.1%), respectively, when the addition content of CNTs was 2.0 wt. %.
(4) The significant enhancement of the yield strength of the nanocomposites was mainly attributed to the efficient load transfer, Orowan mechanism, and thermal mismatch hardening. However, the weakened basal plane texture, CNT clusters distributing on the grain boundaries, micro-pores, and weak interfacial bonding might reduce the strengthening effect of CNTs, which led to discrepancies between the theoretical values and the experimental values.
(5) Adding CNTs could significantly decrease the friction coefficient and weight loss of the matrix due to its self-lubrication effect and the formation of the carbon film covering the wear surface. The relationships between the amount of CNTs and the friction coefficient and weight loss could be described by the exponential decay model and Boltzmann model, respectively.

## Figures and Tables

**Table 1:** Purity and particle sizes of metal powders used in this study.

Materials	Mg	Al	Zn
Purity (%)	>99.9	>99.8	>99.8
Particle size (μm)	45	10	10

**Table 2:** Theoretical and experimental densities of AZ31 and its nanocomposites. CNTs: carbon nanotubes.

Material	Theoretical Density (g/cm^3^)	Experimental Density (g/cm^3^)	Porosity (%)
AZ31	1.7703	1.7637 ± 0.0011	0.4 ± 0.06
AZ31-0.5 wt. % CNTs	1.7719	1.7327 ± 0.0017	2.2 ± 0.09
AZ31-1.0 wt. % CNTs	1.7734	1.7291 ± 0.0035	2.5 ± 0.20
AZ31-2.0 wt. % CNTs	1.7766	1.7298 ± 0.0013	2.6 ± 0.07
AZ31-4.0 wt. % CNTs	1.8136	1.7173 ± 0.0024	5.3 ± 0.13

**Table 3:** Texture results of AZ31 and its nanocomposites (taken perpendicular to the extrusion direction) based on X-ray diffraction.

Material	Plane		I/Imax
AZ31	{0002}	Basal	0.10
	$\left\{10\bar{1}0\right\}$	Prism	1.00
	$\left\{10\bar{1}1\right\}$	Pyramidal	0.67
AZ31-0.5 wt. % CNTs	{0002}	Basal	0.04
	$\left\{10\bar{1}0\right\}$	Prism	1.00
	$\left\{10\bar{1}1\right\}$	Pyramidal	0.49
AZ31-1.0 wt. % CNTs	{0002}	Basal	0.03
	$\left\{10\bar{1}0\right\}$	Prism	1.00
	$\left\{10\bar{1}1\right\}$	Pyramidal	0.69
AZ31-2.0 wt. % CNTs	{0002}	Basal	0.04
	$\left\{10\bar{1}0\right\}$	Prism	1.00
	$\left\{10\bar{1}1\right\}$	Pyramidal	0.97
AZ31-4.0 wt. % CNTs	{0002}	Basal	0.04
	$\left\{10\bar{1}0\right\}$	Prism	1.00
	$\left\{10\bar{1}1\right\}$	Pyramidal	0.94

**Table 4:** Mechanical properties of the as-extruded AZ31 and its nanocomposites.

Material	YS (MPa)	UTS (MPa)	Elongation (%)		Microhardness (HV)
AZ31	188 ± 5.2	280 ± 4.5	14.4 ± 1.2	81.1 ± 1.1	
AZ31-0.5 wt. % CNTs	215 ± 3.5	291 ± 3.8	13.3 ± 1.8	85.5 ± 2.1	
AZ31-1.0 wt. % CNTs	235 ± 4.8	293 ± 5.5	10.9 ± 1.1	87.2 ± 1.8	
AZ31-2.0 wt. % CNTs	241 ± 4.3	297 ± 3.6	9.2 ± 2.0	81.4 ± 1.7	
AZ31-4.0 wt. % CNTs	228 ± 6.5	283 ± 5.8	6.9 ± 2.8	78.3 ± 1.4	

**Table 5:** Regression equations of the friction coefficient versus the content of CNTs (wt. %).

No.	Load (N)	Equations	R-Square
1	5	$\mu_{5N}=0.39+\frac{0.12}{1+\exp(1.86W_{CNT}-2.56)}$	0.9940
2	10	$\mu_{10N}=0.38+\frac{0.12}{1+\exp(1.85W_{CNT}-2.63)}$	0.9992
3	50	$\mu_{50N}=0.34+\frac{0.53}{1+\exp(0.77W_{CNT}-0.97)}$	0.9762

**Table 6:** Regression equations of the mass loss versus the content of CNTs (wt. %).

No.	Load (N)	Equations	R-Square
1	5	$\mu_{5N}=0.39+\frac{0.12}{1+\exp(1.86W_{CNT}-2.56)}$	0.97141
2	10	$\mu_{10N}=0.38+\frac{0.12}{1+\exp(1.85W_{CNT}-2.63)}$	0.9595
3	50	$\mu_{50N}=0.34+\frac{0.53}{1+\exp(0.77W_{CNT}-0.97)}$	0.9657

## References

[1] Pollock T.M. (2010). Weight Loss with Magnesium Alloys. Science.

[2] Schmale J., Shindell D., Schneidemesser E.V., Chabay I., Lawrence M. (2014). Clean up our skies. Nature.

[3] Mcnutt M. (2013). Climate Change Impacts. Science.

[4] Nie J.F., Fang X.Y. (2013). Periodic segregation of solute atoms in fully coherent twin boundaries. Science.

[5] Mirza F., Chen D.L. (2015). A Unified Model for the Prediction of Yield Strength in Particulate-Reinforced Metal Matrix Nanocomposites. Materials.

[6] Miracle D.B. (2005). Metal matrix composites–From science to technological significance. Compos. Sci. Technol..

[7] Iijima S. (1991). Helical microtubules of graphitic carbon. Nature.

[8] Sun F.J., Shi C., Rhee K.Y., Zhao N.Q. (2013). In situ synthesis of CNTs in Mg powder at low temperature for fabricating reinforced Mg composites. J. Alloys Compd..

[9] Merino C.A., Sillas J.E.L., Meza J.M., Ramirez J.M.H. (2016). Metal matrix composites reinforced with carbon nanotubes by an alternative technique. J. Alloys Compd..

[10] Han G.Q., Wang Z.H., Liu K., Li S.B., Du X., Du W.B. (2015). Synthesis of CNT-reinforced AZ31 magnesium alloy composites with uniformly distributed CNTs. Mater. Sci. Eng. A.

[11] Agarwal A., Bakshi S.R., Lahiri D. (2010). Carbon Nanotubes: Reinforced Metal Matrix Composites.

[12] Habibi M.K., Hamouda A.M.S., Gupta M. (2012). Enhancing tensile and compressive strength of magnesium using ball milled Al+CNT reinforcement. Compos. Sci. Technol..

[13] Li C.D., Wang X.J., Liu W.Q., Shi H.L., Ding C., Hu X.S., Zheng M.Y., Wu K. (2014). Effect of solidification on microstructures and mechanical properties of carbon nanotubes reinforced magnesium matrix composite. Mater. Des..

[14] Goh C.S., Wei J., Lee L.C., Gupta M. (2008). Ductility improvement and fatigue studies in Mg-CNT nanocomposites. Compos. Sci. Technol..

[15] Tjong S.C. (2013). Recent progress in the development and properties of novel metal matrix nanocomposites reinforced with carbon nanotubes and graphene nanosheets. Mater. Sci. Eng. R.

[16] Agarwal A., Bakshi S.R., Lahiri D. (2010). Carbon Nanotubes: Reinforced Metal Matrix Composites.

[17] Zeng X.S., Liu Y., Huang Q.Y., Zeng G., Zhou G.H. (2013). Effects of carbon nanotubes on the microstructure and mechanical properties of the wrought Mg–2.0Zn alloy. Mater. Sci. Eng. A.

[18] Bakshi S.R., Lahiri D., Agarwal A. (2010). Carbon nanotube reinforced metal matrix composites-a review. Int. Mater. Rev..

[19] Shimizu Y., Miki S., Soga T., Itoh I., Todoroki H., Hosono T., Sakaki K., Hayashi T., Kim Y.A., Endo M. (2008). Multi-walled carbon nanotube-reinforced magnesium alloy composites. Scr. Mater..

[20] Yuan Q.H., Zeng X.S., Liu Y., Luo L., Wu J.B., Wang Y.C., Zhou G.H. (2016). Microstructure and mechanical properties of AZ91 alloy reinforced by carbon nanotubes coated with MgO. Carbon.

[21] Li C.D., Wang X.J., Liu W.Q., Wu K., Shi H.L., Ding C., Hu X.S., Zheng M.Y. (2014). Microstructure and strengthening mechanism of carbon nanotubes reinforced magnesium matrix composite. Mater. Sci. Eng. A.

[22] Mokdad F., Chen D.L., Liu Z.Y., Xiao B.L., Ni D.R., Ma Z.Y. (2016). Deformation and strengthening mechanisms of a carbon nanotube reinforced aluminum composite. Carbon.

[23] Ferguson J.B., Sheykh-Jaberi F., Kim C.S., Rohatgi P.K., Cho K. (2012). On the strength and strain to failure in particle-reinforced magnesium metal-matrix nanocomposites (Mg MMNCs). Mater. Sci. Eng. A.

[24] Kelly A., Tyson W.R., Mech J. (1965). Tensile properties of fibre-reinforced metals: Copper/tungsten and copper/molybdenum. Phys. Solids.

[25] George R., Kashyap K.T., Rahul R., Yamdagni S. (2005). Strengthening in carbon nanotube/aluminium (CNT/Al) composites. Scr. Mater..

[26] Miller W.S., Humphreys F.J. (1991). Strengthening mechanisms in particulate metal-matrix composites: Reply to comments by Arsenault. Scr. Mater..

[27] Li Q.Q., Viereckl A., Rottmair C.A., Singer R.F. (2009). Improved processing of carbon nanotube/magnesium alloy composites. Compos. Sci. Technol..

[28] Frost H.J., Ashby M.F. (1982). Deformation Mechanism Maps: The Plasticity and Creep of Metals and Ceramics.

[29] Rashad M., Pan F.S., Tang A., Asif M. (2014). Effect of Graphene Nanoplatelets addition on mechanical properties of pure aluminum using a semi-powder method. Prog. Nat. Sci..

[30] Li Q.Q., Rottmair C.A., Singer R.F. (2010). CNT reinforced light metal composites produced by melt stirring and by high pressure die casting. Compos. Sci. Technol..

[31] Zhang Z., Chen D.L. (2006). Consideration of Orowan strengthening effect in particulate-reinforced metal matrix nanocomposites: A model for predicting their yield strength. Scr. Mater..

[32] Goh C.S., Wei J., Lee L.C., Gupta M. (2006). Development of novel carbon nanotube reinforced magnesium nanocomposites using the powder metallurgy technique. Nanotechnology.

[33] Liu J.L., Zhao K., Zhang M., Wang Y.G., An L.N. (2015). High performance heterogeneous magnesium-based nanocomposite. Mater. Lett..

[34] Bartolucci S.F., Paras J., Rafiee M.A., Rafiee J., Lee S., Kapoor D., Koratkar N. (2011). Graphene-aluminum nanocomposites. Mater. Sci. Eng. A.

[35] Suryanarayana C., Ivanov E., Boldyrev V.V. (2001). The science and technology of mechanical alloying. Mater. Sci. Eng. A.

[36] Rashad M., Pan F.S., Zhang J., Asif M. (2015). Use of high energy ball milling to study the role of graphene nanoplatelets and carbon nanotubes reinforced magnesium alloy. J. Alloys Compd..

[37] Rashad M., Pan F.S., Hu H., Asif M., Hussain S., She J. (2015). Enhanced tensile properties of magnesium composites reinforced with graphene nanoplatelets. Mater. Sci. Eng. A.

[38] Guo L., Chen Z., Gao L. (2011). Effects of grain size, texture and twinning on mechanical properties and work-hardening behavior of AZ31 magnesium alloys. Mater. Sci. Eng. A.

[39] Hassan H.A., Lewandowski J.J. (2014). Effects of particulate volume fraction on cyclic stress response and fatigue life of AZ91D magnesium alloy metal matrix composites. Mater. Sci. Eng. A.

[40] Wang X.J., Hu X.S., Wu K., Wang L.Y., Huang Y.D. (2015). Evolutions of microstructure and mechanical properties for SiCp/AZ91 composites with different particle contents during extrusion. Mater. Sci. Eng. A.

[41] Ryu H., Cha S., Hong S. (2003). Generalized shear-lag model for load transfer in SiC/Al metal-matrix composites. J. Mater. Res..

[42] Liu Z.Y., Xiao B.L., Wang W.G., Ma Z.Y. (2014). Analysis of carbon nanotube shortening and composite strengthening in carbon nanotube/aluminum composites fabricated by multi-pass friction stir processing. Carbon.

[43] Bakshi S.R., Agarwal A. (2011). An analysis of the factors affecting strengthening in carbon nanotube reinforced aluminum composites. Carbon.

[44] Li C.D., Wang X.J., Wu K., Liu W.Q., Xiang S.L., Ding C., Hu X.S., Zheng M.Y. (2014). Distribution and integrity of carbon nanotubes in carbon nanotube/magnesium composites. J. Alloys Compd..

[45] Chen B., Shen J., Ye X., Imai H., Umeda J., Takahashi M., Kondoh K. (2016). Solid-state interfacial reaction and load transfer efficiency in carbon nanotubes (CNTs)-reinforced aluminum matrix composites. Carbon.

[46] Liu Z.Y., Xiao B.L., Wang W.G., Ma Z.Y. (2012). Singly dispersed carbon nanotube/aluminum composites fabricated by powder metallurgy combined with friction stir processing. Carbon.

[47] Lenel F.V. (1980). Powder Metallurgy Principles and Application.

[48] Choi H.J., Kwon G.B., Lee G.Y., Bae D.H. (2008). Reinforcement with carbon nanotubes in aluminum matrix composites. Scr. Mater..

[49] Shi H.L., Wang X.J., Li C.D., Hu X.S., Ding C., Wu K., Huang Y.D. (2014). A Novel Method to Fabricate CNT/Mg-6Zn Composites with High Strengthening Efficiency. Acta Metall. Sin..

[50] Wu J.B., Zeng X.S., Luo L., Yuan Q.H. (2015). Friction and wear properties of Carbon Nanotubes/AZ91 composites. Mater. Mech. Eng..

[51] Kim I.Y., Lee J.H., Lee G.S., Baik S.H., Kim Y.J., Lee Y.Z. (2009). Friction and wear characteristics of the carbon nanotube–aluminum composites with different manufacturing conditions. Wear.

[52] Scharf T.W., Neira A., Hwang J.Y., Tiley J., Banerjee R. (2009). Self-lubricating carbon nanotube reinforced nickel matrix composites. J. Appl. Phys..

[53] Dong S.R., Tu J.P., Zhang X.B. (2001). An investigation of the sliding wear behavior of Cu-matrix composite reinforced by carbon nanotubes. Mater. Sci. Eng. A.

